# Evaluation of lateral flow assay as a field test for investigation of brucellosis outbreak in an organized buffalo farm: A pilot study

**DOI:** 10.14202/vetworld.2015.492-496

**Published:** 2015-04-16

**Authors:** R. Shome, G. Filia, B. S. Padmashree, N. Krithiga, Swati Sahay, K. Triveni, B. R. Shome, V. Mahajan, Amarjit Singh, H. Rahman

**Affiliations:** 1 Bacteriology Lab-1, National Institute of Veterinary Epidemiology and Disease Informatics (Formerly PD_ADMAS), Ramagondanahalli, Yelahanka, Bengaluru - 560 064, Karnataka, India; 2 Animal Disease Research Centre, College of Veterinary Science, Guru Angad Dev Veterinary and Animal Sciences University, Ludhiana - 141 004, Punjab, India

**Keywords:** brucellosis outbreak, competitive enzyme-linked immunoassay, indirect enzyme-linked immunoassay, lateral flow assay, rose bengal plate test

## Abstract

**Aim:**

The aim was to evaluate lateral flow assay (LFA) as a field test for investigation of brucellosis outbreak in organized buffalo farm.

**Materials and Methods:**

A total of 153 serum samples were tested to detect the presence of *brucella* antibodies by LFA and three other serological tests i.e. rose bengal plate test (RBPT), protein G based indirect enzyme-linked immunoassay (iELISA), and competitive ELISA (cELISA). The performances of LFA and other serological tests were evaluated using OIE complaint cELISA as the gold standard.

**Results:**

Serological tests revealed 50% of the animals were seropositive for *Brucella* antibodies and correlated with clinical history of abortions, infertility, and productive failures. The newly developed assay showed 87.1% and 92.6% sensitivity and specificity, which was even higher than the specificity of RBPT.

**Conclusions:**

The investigation proved the potential usefulness of LFA for field diagnosis of brucellosis in the regions where laboratory facilities are limited.

## Introduction

India has a vast resource of livestock and dairy farming plays a significant role in the country’s rural economy [1]. The country holds the largest buffalo population in the world (105.34 millions - 57.3%) followed by the 2^nd^ largest cattle population (199.08 millions - 14.7%) [2], and highest milk production in the world i.e. 121.8 million tonnes with per capita availability of 281 g/day [3].

Brucellosis is a highly contagious disease of dairy animals and humans in many parts of the World including India causing significant morbidity and enormous economic losses [4,5]. The disease causes abortions in the last trimester of pregnancy, premature births followed by retention of placenta, metritis, decreased milk production and lameness as common sequelae to infection in dairy animals [6]. A national survey in bovines a decade back indicated 5% of cattle and 3% of buffaloes of the country were infected with brucellosis [7]. The occurrence of the disease is usually high in organized farms (50%) compared to the marginal herds (10%) and this primarily associated with intensive farming practices in large organized animal farms [8].

The importance of brucellosis is regionally overlooked and most of the cases were under-diagnosed or misdiagnosed due to non-pathognomonic nature of clinical presentations and lack of simple, reliable, and cost-effective field-based diagnostic tool for early investigation of infection [8]. Even though, several diagnostic approaches like isolation, rose bengal plate agglutination test (RBPT), serum agglutination test, complement fixation test, enzyme-linked immunosorbent assay (ELISA), polymerase chain reaction (PCR), native hapten gel precipitation tests, brucellin skin test, and fluorescence polarization assay were available for brucellosis. All these tests are laboratory-based, requires technical skill, refrigeration, specific equipment, and only a few reference laboratories for brucellosis diagnosis were available in the country. Thus, availability of simple, reliable, cost-effective and field-based diagnostic tool without any requirement of technical skill, refrigeration, and equipment helps to diagnose every reproductive and productive failure due to brucellosis cases in the farm so that positive reactors can be easily identified and separated from other farm animals. This strikingly demands development of a lateral flow assay (LFA), a field-based diagnostic tool for easy detection of infection and to prevent disease transmission and outbreak events. In addition, it also facilitates improvement of current understanding of epidemiology and can serve as important tool for disease surveillance system.

Any newly developed diagnostic test needs to be evaluated with other widely used standard tests to determine its performance accuracy. Thus, the present pilot study was carried out to evaluate the utility of in-house developed LFA along with other routinely used laboratory tests for an investigation of brucellosis outbreak in organized buffalo farm in North India.

## Materials and Methods

### Ethical approval

The present study was approved by Institutional Animal Ethics Committee, ICAR- NIVEDI. The authors have taken permission from farm owner to publish data.

### Herd details

The murrah breed buffalo population at the farm during sample collection was 153 and the animals were maintained as per their physiological status. They were stall-fed with green maize and dry wheat fodder, mineral mixtures and concentrates, and artificial insemination was the breeding method followed. Farm records revealed that there was an outbreak of abortions with retention of placenta in the last trimester of pregnancy from past 7 to 8 months. All the abortions were recorded in second lactation onwards in the age group of 3-10 year animals. The number of abortions increased from 4-5 to 5-8 per month in the farm during later periods which compelled farmer to seek laboratory investigation. The animals were previously vaccinated against foot and mouth disease and hemorrhagic septicemia. However, the animals were neither vaccinated against brucellosis nor previously tested for brucellosis.

### Sample collection

Approximately, 10 ml of the blood sample was collected from the jugular vein of each animal using vacutainers without ethylenediaminetetraacetic acid (Becton Dickson, UK). Serum was separated from clotted blood in vacutainer after 4 h and stored at 20°C.

### Field diagnosis of samples by LFA

**Figure-1:**
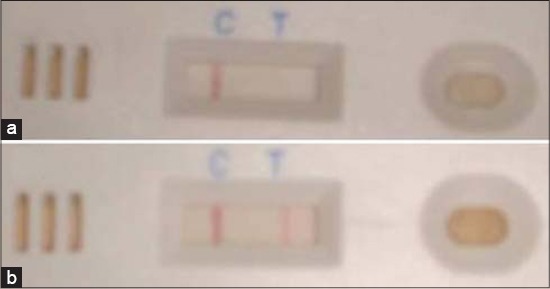
LFA showing test line negative (A) and positive (B)

In-house developed LFA were used for diagnosis. LFA cassettes were developed in collaboration with M/s Ubio Biotechnology Systems Pvt. Ltd., XII-111-E/F, Technology Incubation Centre, KINFRA Hi-Tech Park, Kalamasser, Cochin, Kerala, India - 683 503, using smooth lipopolysaccharide (sLPS) extracted from *Brucella abortus* S99 (procured from National Culture Repository, Indian Veterinary Research Institute, Izatnagar, India). The LFA is a simplified format of ELISA for the qualitative detection of antigen-specific antibodies in serum or whole blood samples. The assay is based on the binding of specific antibodies to an antigen (sLPS) immobilized on a test strip and bound antibodies are visualized using a secondary antibody conjugated to colloidal gold particles. Approximately, 5-10 µl of serum sample was added to the sample port, followed by addition of 2-3 drops of assay diluent and results were recorded within 5-7 min. Appearance of only control line was noted as a test negative and test positive status was recorded when both control and test lines appeared in test zone of the device (Figure-1).

First stage evaluation of LFA test for a panel of 200 bovine serum samples (100 each of cattle and buffalo) showed kappa coefficient of 0.9 with RBPT and iELISA. Hence, LFA devices were used in this pilot study for field evaluation.

Laboratory diagnosis of samples by different serological tests: All the serum (n=153) samples were analyzed by RBPT according to standard protocol [9] with the *B. abortus* S99 colored antigen procured from Institute of Animal Health and Veterinary Biologicals, Hebbal, Bangalore, India. And then, iELISA was performed using sLPS antigen from standard strain *B. abortus* 99 as per the Office International des Epizooties (OIE) protocol [10]. The cut-off values established for the diagnosis was decided after thorough screening and validation of assay [11]. Any sample of percent positivity (PP) value below 55% is taken as negative, between 55% and 65% as moderate positive, more than 65% as strong positive and sample with only 55% PP are recommended for retesting for confirmation. OIE complaint competitive ELISA (cELISA) was performed as per the manufacturer’s protocol (LT Biotech).

### Statistical analysis

The results of RBPT, iELISA, and LFA were evaluated in comparison with cELISA as the gold standard due to its high specificity and sensitivity [12]. Sensitivity and specificity of each test were calculated using Med Calc statistical software (http://www.medcalc.org/calc/diagnostic_test.php).

## Results

Test wise analysis revealed that out of 153 animals tested, 85 (55.5%), 83 (54.2%), 82 (53.5%), and 79 (51.6%) were positive by c ELISA, iELISA, RBPT, and LFA, respectively. Disease status of the animals and comparison of diagnostic assays were interpreted by cELISA as the gold standard in the absence of isolation. Age-wise analysis revealed that the highest sero-prevalence was recorded in the age group of 4.1-8 years (74.7%) followed by more than 8 years (66.7%) and lowest in below 2 years age group (14.2%). Of the 54 pregnant animals in the farm, 51 (94.4%) were seropositive and among heifer and milch groups, 2 (14.2%), and 32 (37.6%) animals were positive, respectively. Similarly, 40 (100%), 14 (48.2), 10 (45.4), and 21 (33.8) buffaloes were positive in groups with clinical history of abortion, infertility, reduced milk production, and no visible clinical signs, respectively (Table-1).

**Table-1:** Age, physiological status and clinical history wise prevalence of brucellosis in buffalo farm.

Test positives versus groups	Age (years)				Physiological status			Clinical history				Test
			
	1-2	2.1-4	4.1-8	>8	Heifers	Milch animals	Pregnant animals**	Abortion	RB and infertility	Reduced milk production	No clinical symptoms	Total
Number of animals	14 (9.1)*	33 (21.5)	91 (59.4)	15 (9.8)	14 (9.1)	85 (55.5)	54 (35.2)	40 (26.1)	29 (18.9)	22 (14.3)	62 (54.9)	153
Field diagnosis of samples by LFA												
LFA	02 (14.2)	04 (12.1)	65 (71.4)	08 (53.3)	02 (14.2)	30 (35.2)	47 (87.0)	40 (100.0)	13 (44.82)	9 (40.1)	17 (27.4)	79 (51.6)
Laboratory diagnosis of samples by other tests												
RBPT	01 (7.1)	05 (15.1)	67 (73.6)	09 (60.0)	01 (7.1)	31 (36.4)	50 (92.5)	40 (100.0)	12 (41.3)	9 (40.1)	21 (33.8)	82 (53.5)
iELISA	01 (7.1)	05 (15.1)	68 (74.7)	09 (60.0)	01 (7.1)	31 (36.4)	51 (94.4)	40 (100.0)	13 (44.8)	9 (40.1)	21 (33.8)	83 (54.2)
cELISA	02 (14.2)	05 (15.1)	68 (74.7)	10 (66.7)	02 (14.2)	32 (37.6)	51 (94.4)	40 (100.0)	14 (48.2)	10 (45.4)	21 (33.8)	85 (55.5)

LFA=Lateral flow assay, RBPT=Rose Bengal plate test, iELISA=Indirect enzyme-linked immunoassay, cELISA=Competitive enzyme-linked immunoassay,

* Values are represented in terms of a percentage; RB-repeat breeding.

** Total 54 of includes 40 aborted, 14 pregnant animals

The sensitivity and specificity of LFA versus cELISA were found to be 87.1% and 92.6%, respectively. The positive predictive value (PPV) and negative predictive value (NPV) of the test were found to be 93.7% and 85.1%, respectively (Table-2). The highest sensitivity and specificity were recorded with in-house developed iELISA (94.1% and 95.6%) and lowest with RBPT (91.5% and 85.9%).

**Table-2:** Evaluation of LFA, RBPT, and iELISA in comparison with cELISA as the gold standard.

Diagnostic tests	LFA	RBPT	iELISA
PPV	93.7 (85.8-97.8)	91.5 (83.2-96.5)	96.4 (89.7-99.2)
NPV	85.1 (74.9-92.4)	85.9 (75.6-93.0)	92.9 (84.1-97.6)
Sensitivity	87.1 (78.0-93.3)	88.2 (79.4-94.2)	94.1 (86.8-99.0)
Specificity	92.6 (83.7-97.5)	89.7 (79.9-95.7)	95.6 (87.6-99.0)
Positive likelihood ratio	11.8 (5.1-27.6)	8.6 (4.2-17.4)	21.4 (7.0-64.5)
Negative likelihood ratio	0.14 (0.1-0.2)	0.13 (0.1-0.2)	0.06 (0.03-0.1)

*PPV=Positive predictive value, NPV=Negative predictive value, Values are represented in terms of percentage; Values in parenthesis represent 95% Confidential Interval (CI)

## Discussion

Brucellosis is a chronic bacterial disease causing huge economic losses to the livestock industry not only in terms of abortion and other reproductive problems, but also associated with lower milk yields (20-25%) [13]. In buffalo herds, abortions followed by retained placenta can be seen as one of the most obvious signs, generally evident in the last third of gestation [14]. The overall prevalence of the disease was found very high in buffaloes by all serological tests (>50%) in the present outbreak. It is important to note that 21 out of 62 buffaloes (33.8%) with no visible clinical signs were seropositive. Significantly, 51 out of 54 pregnant animals were also seropositive and this undoubtedly demonstrated the severity of infection in buffalos. The similar reports of high sero-positivity in farms with reduced productive and reproductive conditions have been reported [15-18]. In India, seroprevalence up to 41.6% and 50% has been recorded in cattle and buffaloes, respectively with history of repeat breeding and abortion [19]. The disease prevalence in different age groups, when compared, high seroprevalence was recorded in the age group of 4.1-8 years (74.7%) and more than 8 years (66.7%). The susceptibility to brucellosis increases with age and more commonly associated with sexual maturity than age [20]. Few sero-positives cases detected in below 2 year age group of animals (14.2%) may be due to exposure to brucellosis infected animals in the farms. Among milch animals, 32 out of 85 (37.6%) were positive. It has been reported from several studies that, excretion of an organism through milk possess a great risk of zoonotic transmission of infection to humans. These alarms need for routine milk diagnosis at least twice a year by milk ring test for health concern of farm animals and consumers.

Brucellosis causes abortions in farm animals and infected animals continue to act as source of infection [21]. The high infection status in the herd is attributed to frequent abortions and transmission to other buffaloes in the herd environment. The other reason could be due to lack of diagnosis prior to the introduction of new animal into the farm and at the time of every abortion event. Overcrowding with inadequate floor space and unsatisfactory sanitation were other potential causes facilitated the disease transmission. Thus, prior diagnosis before introduction of new animal in organized farms assumes paramount importance in brucellosis control. The diagnostic test used for this purpose should be quick, handy, cost-effective, and reasonably sensitive for regular brucellosis screening in the herds. In India, RBPT is widely used for its simplicity [22-24], whereas ELISA is commonly used in surveillance which requires technical skill and equipment and hence it is not suitable for field diagnosis [25]. The RBPT test is often compounded with false positive results due its low specificity [25]. Hence, there is a great demand in the country for a simple test for the field use with similar and or higher sensitivity and specificity than RBPT. In addition, LFA as a field test requires neither specific expertise nor equipment and test devices may be kept in stock without refrigeration [26]. Hence, in the present investigation, LFA was evaluated with RBPT and iELISA considering cELISA as the gold standard. Our results are in agreement with the high sensitivity and specificity observed for the flow assay by earlier reports from Spain [27], Turkey [28,29] Kazakhstan [28], and Egypt [30,31]. The LFA has shown good PPV and NPV greater than RBPT and almost similar to that of iELISA. Even though the sensitivity of LFA is lower than that of ELISA, it is almost closer to that of RBPT with specificity higher than RBPT. The higher specificity is optimal for minimizing the false positive results in the field conditions and proves that the LFA is a good test for sero-diagnosis in the field.

The LFA is probably not ideal for large-scale screening, but could be a very useful tool to identify infected animals in smallholder herds, so that they can be removed or their milk is rejected or for providing public health advice to farmers following abortions in their herds. In general, serological methods used solitary carry the risk to interpret false negative results. Therefore, use of RBPT and the flow assay, or a combination of the two tests appears a good choice for countries such as India where brucellosis is endemic, but laboratory support is not readily available. This study also demonstrates the potential usefulness of this simple test to use in field based surveillance, which could be easily adopted without basic laboratory facilities.

## Conclusion

Brucellosis is a well-known cause of reproductive and productive losses in ruminants. Diagnosis of infected animals and removing from the herd is a key to the control the disease in livestock and the human population. In endemic countries, the diagnosis is frequently missed because laboratory facilities are poorly equipped and “modern” diagnostic means such as ELISA and PCR are considered too expensive. In view of these, the authors try to provide solutions and recommendations for translational research regarding the use of novel diagnostics such as LFA. The LFA has shown good PPV and NPV greater than RBPT in the current study suggest that the test is a simple, cost-effective and rapid that provides accurate detection of antibodies to *B. abortus* in bovine serum samples, thereby saving time and eliminating the need for special training. This rapid test can therefore be practically implemented in serological screening for bovine brucellosis, although evaluation on a larger scale with various cattle sera, and blood samples is still necessary.

## Authors’ Contributions

The present study was a part of DBT-Network Project on Brucellosis. RS conceptualized the aim of the study, designed, planned, and supervised the experiments and corrected the manuscript. BSP has formulated objectives of the study, conducted the experimental analysis of samples, statistical analysis of data, interpretation of the results, and drafting of the manuscript. NK, SS, and KT have supported execution of experiments and drafting of the manuscript. AS, VM, and GF helped in collection and dispatch of samples along with sample history. BRS and HR helped in the revision of the manuscript. All authors read and approved the final manuscript.

## References

[1] Kumar R, Prabhakar R.K (2013). Opportunities and challenges in Indian dairy industry supply chain: A literature review. Int. J. Logist. Suppl. Chain. Manage. Perspect.

[2] Livestock Census (2007). Department of Animal Husbandry, Dairying and Fisheries, Ministry of Agriculture, Government of India.

[3] Government of India (2012). Basic Animal Husbandry Statistics. Department of Animal Husbandry, Dairying and Fisheries.

[4] Singh G, Sharma D.R, Sandhu K.S, Dhand N.K (2002). Economic losses occurring due to bovine abortions in Punjab. 10th International Congress of Asian-Australasian Association of Animal Production Societies, 23-27 September, New Delhi, India.

[5] McDermott J, Grace D, Zinsstag J (2013). Economics of brucellosis impact and control in low-income countries. Rev. Sci. Tech.

[6] Megid J, Mathias L.A, Robles C.A (2010). Clinical manifestations of brucellosis in domestic animals and humans. Open Vet. Sci. J.

[7] Renukaradhya G.J, Isloor S, Rajasekhar M (2002). Epidemiology, zoonotic aspects, vaccination and control/eradication of brucellosis in India. Vet. Microbiol.

[8] Smits H.L, Kadri S.M (2005). Brucellosis in India: A deceptive infectious disease. Indian J. Med. Res.

[9] Alton G.G, Jones L.M, Angus R.D, Verger J.M (1988). Techniques for the Brucellosis Laboratory.

[10] World Organization for Animal Health (2012). Manual of Diagnostic Tests and Vaccines for Terrestrial Animals.

[11] Shome R, Gangadar N.L, Narayana Rao K, Shome B.R, Prabhudas K (2011). Diagnosis of brucellosis in the equines by serological tests and PCR: A clinical report. Indian. J. Anim. Sci.

[12] Lorraine L.P, John A.M, Simon D.B, Judith A.S (2005). Evaluation of competitive ELISA for detection of antibodies to *Brucella* infection in domestic animals. Clin. Diagn. Lab. Immunol.

[13] ILRI (2012). Mapping of poverty and likely zoonoses hotspots.

[14] Shafee M, Masood R, Ali A.S, Mansoor D.A, Abdul R (2011). Prevalence of bovine brucellosis in organized dairy farms, using milk ELISA, in Quetta city, Balochistan, Pakistan. Vet. Med. Int.

[15] Bekele A, Molla B, Asfaw Y, Yigezu L (2000). Bovine brucellosis in ranches and farms in southeastern Ethiopia. Bull. Anim. Health. Prod. Afr.

[16] Silva I, Dangolla A, Kulachelvy K (2000). Seroepidemiology of *Brucella abortus* infection in bovids in Sri Lanka. Prev. Vet. Med.

[17] Amin K.M, Rahman M.B, Rahman M.S, Han J, Park J.H, Chae J.S (2005). Prevalence of *Brucella* antibodies in sera of cows in Bangladesh. J. Vet. Sci.

[18] Kushwaha N, Rajora V.S, Mohan A, Nadeem M, Arora N (2013). Prevalence of bovine brucellosis in a dairy herd, Uttarakhand, India. Indian J. Anim. Sci.

[19] Pandey S, Chachra D, Kaur P, Chandra M, Sharma N.S (2014). Conventional and molecular characterization of *Brucella abortus* and detection of antibodies against *Brucella abortus* in cattle and buffaloes. Indian J. Anim. Sci.

[20] Radostits O.M, Gay C.C, Blood D.C, Hinchcliff K.W (2000). Veterinary Medicine.

[21] European Commission (2001). Scientific committee on animal health and animal welfare *Brucella melitensis* in sheep and goats.

[22] Gall D, Nielsen K (2004). Serological diagnosis of bovine brucellosis: A review of test performance and cost comparison. Rev. Sci. Tech.

[23] Muma J.B, Toft N, Oloya J, Lund A, Nielsen K, Samui K, Skjerve E (2007). Evaluation of three serological tests for brucellosis in naturally infected cattle using latent class analysis. Vet. Microbiol.

[24] Emmerzaal A, Dewit J.J, Dijkstran T, Bakker D, Van Zijderveld F.G (2002). The Dutch *Brucella abortus* monitoring programme for cattle: the impact of false positive serological reactions and comparison of serological tests. Vet. Q.

[25] Gwida M.M, El-Gohary A.H, Melzer F, Tomaso H, Rosler U, Wernery U, Wernery R, Elschner M.C, Khan I, Eickhoff M, Schöner D, Neubauer H (2011). Comparison of diagnostic tests for the detection of *Brucella* spp.in camel sera. BMC Res Notes.

[26] Bronsvoort B.M.D, Koterwas B, Land F, Handel I.G, Tucker J, Morgan K.L, Tanya V.N, Abdoel T.H, Smits H.L (2009). Comparison of a flow assay for brucellosis antibodies with the reference cELISA test in West African *Bos indicus*. Plos One.

[27] Smits H.L, Abdoel T.H, Solera J, Clavijo E, Diaz R (2003). Immunochromatographic *Brucella-* Specific Immunoglobulin M and G lateral flow assays for rapid serodiagnosis of human brucellosis. Clin. Diagn. Lab. Immunol.

[28] Irmak H, Buzgan T, Evirgen O, Akdeniz H, Demiroz A.P, Abdoel T.H, Smits H.L (2004). Use of the *Brucella* IgM and IgG flow assays in the serodiagnosis of human brucellosis in an area endemic for brucellosis. Am. J. Trop. Med. Hyg.

[29] Zeytinoglu A, Turhan A, Altuglu I, Bilgic A, Abdoel T.H, Smits H.L (2006). Comparison of *Brucella* immunoglobulin M and G flow assays with serum agglutination and 2-mercaptoethanol tests in the diagnosis of brucellosis. Clin. Chem. Lab. Med.

[30] Marei A, Boghdadi G, Abdel-Hamed N, Hessin R, Abdoel T, Smits H, Fathey F (2011). Laboratory diagnosis of human brucellosis in Egypt and persistence of the pathogen following treatment. J. Infect. Dev. Ctries.

[31] Elshemey T.M, Abd-Elrahman A.H (2014). Evaluation of a Rapid Immunochromatographic test for detection of *Brucella abortus* antibodies in Egyptian cattle sera and milk. Alex. J. Vet. Sci.

